# Nutritional optic and peripheral neuropathy: a case report

**DOI:** 10.4076/1757-1626-2-7762

**Published:** 2009-06-05

**Authors:** Laura M Nightingale, Dominic C Paviour

**Affiliations:** 1 A&E Department, St Thomas' HospitalLambeth Palace Road, London SE1 7EHUK; 2 Department of Neurology, St Thomas' HospitalWestminster Bridge Road, London SE1 7EHUK

## Abstract

**Introduction:**

The link between nutritional status and either optic or peripheral neuropathies is well established with tobacco, ethanol, deficiencies in thiamine, vitamin A, B12, B3 and B6 and protein-energy malnutrition all being causative.

**Case presentation:**

We describe the case of a 32-year-old Afro-Caribbean of Jamaican origin presenting with blurred vision and a painful burning sensation in his feet. The clinical features were consistent with optic and peripheral neuropathy.

**Conclusions:**

The patient followed a strict vegan diet and consumed no animal products. A review of the literature highlights similarities between this case and Strachan's syndrome, a combination of optic and peripheral neuropathy and cutaneous excoriation, providing further evidence for the association between this clinical presentation, dietary deficiency and, as recently postulated, previous residence in a tropical or sub-tropical climate.

## Introduction

The link between nutritional status and either optic or peripheral neuropathies is well established with tobacco, ethanol, deficiencies in thiamine, vitamin A, B12, B3 and B6 and protein-energy malnutrition all being causative.

## Case presentation

A 32-year-old Afro-Caribbean male presented with a three-month history of blurred vision and a two-month history of bilateral leg weakness accompanied by a painful burning sensation in his feet and a painful skin rash on the lower limbs.

He had no significant past medical history and had an extensive family comprising of 10 sisters, five brothers and six children (three male, three female) whom he stated were all fit and well.

He took no regular medication and denied any allergies. He was originally from Jamaica and had been resident in the UK for nine years. He denied any recent foreign travel. He did not work and was the sole carer for his six children. He smoked approximately five cigarettes a day, as well as twice-weekly cannabis. He consumed alcohol rarely and denied any further recreational drug use. He was a strict vegan consuming no meat, fish, eggs or dairy products, after adopting the Rastafarian religion one-year previously. His diet consisted of nuts, pulses, seeds, a variety of fruit and vegetables, as well as tofu, soya products, rice, whole-grain bread and cereals, including some fortified products occasionally and vegetable oils.

On presentation, opthalmological examination revealed corneal changes consistent with punctate epitheliopathy. Visual acuity was impaired to the extent of being able only to count fingers at the bedside and was subjectively slightly worse on the right than the left; it did not improve with refraction. Pupils were equal and reactive with a right-sided afferent pupillary defect. Slit lamp examination revealed temporal disc pallor with a pigmentary scar in the right infero-temporal retina. Cranial nerve examination was otherwise unremarkable.

Neurological Examination of the upper limbs was normal. Examination of the lower limbs revealed proximal weakness with preserved reflexes and equivocal plantars. He had subtle impairment of proprioception to the ankle with impaired light touch, vibration and pin prick in a stocking distribution. He walked with an ataxic and antalgic gait.

Dermatological examination revealed a tender nodular eruption with small vesicles on top of some lesions over the lower legs bilaterally. There were no other muco-cutaneous lesions, and cardiovascular, respiratory and abdominal examinations were all unremarkable.

BMI was calculated at 21. Full blood count, urea and electrolytes and liver function tests were normal; haemoglobin was 15.6 (normal 13.0-17.0 g/dl), creatinine 80 (normal 59-104 umol/L), albumin 50 (normal 40-52 g/L) and total protein 67 (normal 60-85 g/L). Coagulation screen was normal with an INR of 0.97 (normal 0.90-1.10), APTT 1.01 (normal 0.8-1.16) and fibrinogen 3.39 (normal 1.67-5.43). Inflammatory and auto-immune profiles (anti-neutrophil antibodies, anti-nuclear antibody, ENA antibody, ESR and CRP) were normal aside from a very weakly positive antinuclear antibody with speckled pattern. Serum vitamin B12 was moderately low at 111 (normal 145-1000 ng/L), folate was normal at 3.8 (normal 2.5-16.0 ug/L). Iron studies were normal; Iron 21.0 (normal 14.0-31.0 umol/L), total iron binding capacity 64 (normal 41-77 umol/L), transferrin saturation 60 (normal 18-71%). Serum calcium was 2.21 corrected (normal 2.15-2.55 mmol/L) and phosphate 1.2 (normal 0.9-1.4 mmol/L). The following investigations were all negative: serum ACE, Treponema pallidum, antibodies and HIV. Goldmann visual field assessment revealed constricted visual fields bilaterally, with a small central scotoma.

An MRI scan of the brain was normal. In particular there was no parenchymal or meningeal enhancement and optic nerve sheath complexes were normal with no contrast enhancement. The cavernous sinuses were normal and there were no retro-orbital mass lesions. MRI of the whole spine revealed no significant abnormality.

There were no features to suggest a motor or sensory neuropathy on electromyography (EMG) or nerve conduction studies, although thermal thresholds to assess for a small fibre neuropathy were not performed.

A dermatological review suggested a folliculitis and skin biopsy was felt to be consistent with this, although changes in the subcutis were indicative of a non-specific lobular panniculitis.

A Schilling test was not performed as the B12 deficiency was thought to be nutritional in origin and full audiological testing was not undertaken as the patient reported no hearing impairment. CSF examination was not performed during this admission and the patient declined genetic testing for Leber's hereditary optic neuropathy (LHON).

A diagnosis of Optic and Peripheral Neuropathy was made, which was thought to be nutritional in origin, and the patient was commenced on Intravenous B vitamins and Vitamin B12 intra-muscular injections. He was discharged home on Folate and Vitamin B Co-Strong tablets, Gabapentin and Amitryptyline for neuropathic pain and Vitamin B 12 intra-muscular injections on alternate days for two weeks and then twice monthly after this.

He was reviewed as an outpatient two months after discharge and he reported no improvement to his vision or lower limb weakness and pain.

## Discussion

In 1887 whilst working as a physician in Jamaica, Dr H Strachan first noticed the concurrent development of blurred vision, painful sensorimotor neuropathy and cutaneous excoriation which later became known as Strachan's disease [1]. Since then, similar symptoms have been reported throughout the West Indies, Cuba, Tanzania, Nigeria and in Prisoner-of war camps in the tropics [2]. Characteristics, which may include sensorineural deafness, mucocutaneous lesions and more commonly a painful sensorimotor neuropathy and visual loss, have come to make up the spectrum known as Strachan's syndrome [3].

These patients commonly show evidence of a bilateral reduction in visual acuity, along with optic disc pallor, optic atrophy and large central scotomas on visual field testing. Neurologically, there is evidence of a sensorimotor neuropathy both clinically and on electromyography (EMG) and nerve conduction studies. Less frequently there is evidence of bilateral sensorineural deafness and mucocutaneous lesions consistent with nutritional deficiency such as glossitis, angular stomatitis and desquamation of the skin [3-5].

The aetiology remains uncertain, and over the years a number of causes have been postulated including the consumption of cyanogenic foods such as Cassava, although the exact mechanism of neurotoxicity was unknown [5]. This theory has subsequently been cast aside and the disease is now believed to be nutritional in origin, resulting from a vitamin deficiency which is distinct from thiamine and pyridoxine which may cause a similar syndrome if deficient [6]. In the majority of cases of Strachan's, assays have been undertaken which have failed to reveal any clinical evidence of specific vitamin deficiencies and the cause is presumed to be nutritional as the majority of people suffering from the disease have been malnourished [3,5,7].

A recent paper casts doubt on a nutritional aetiology, as similar symptoms were reported in two patients, born in the West Indies but long term residents of the UK, both of whom were well nourished with no evidence of vitamin deficiency. The common aetiological thread, it was suggested, was a period of residence in a tropical or sub-tropical climate [7].

Vitamin supplementation following the onset of symptoms has produced varied response, with at best, limited improvement in symptoms [5,7,8]. However, there is some evidence that vitamin supplementation may prevent the occurrence of new cases in areas where the syndrome is prevalent [4].

It is widely recognised that strict vegans and raw food-low fat consumer are at risk of vitamin A and B-12 deficiency with the resultant low vision, temporal blindness, muscle weakness and skin problems which make up a clinical picture similar to that seen in Strachan's [9,10]. Although the patient we describe had moderate Vitamin B12 deficiency the clinical impression was that his symptoms were too severe to be attributable purely to B12 deficiency alone. His failure to respond to multivitamin and specifically B12 supplementation lends further weight to this argument, as in B12 deficiency alone, improvement of symptoms is seen soon after treatment has begun [11]. He reported a good dietary intake of both raw and cooked vegetables, nuts, seeds and vegetable oils, making vitamin A deficiency as a cause of his symptoms very unlikely [10].

Previously, similar clinical presentations have been seen in protein-energy malnutrition, characterised by low fat consumption and sulphur amino acid misbalance [12]. However, despite this patient's strict vegan diet, he had a satisfactory protein intake as reflected in his blood results, and we feel this is unlikely to be the cause of the clinical manifestation in this case.

The similarity of our patient to Strachan's original description is apparent, with the complaint of a painful burning sensation in the feet and the visual loss with accompanied sensorimotor neuropathy. So indeed, is the correlation between the clinical findings in our patient and those reported in the published literature on the subject, and his limited response to vitamin supplementation. To our knowledge, there are no other reports of a panniculitis occurring in combination with the optic and neurological symptoms, and the relevance of this remains uncertain.

**Figure 1.**
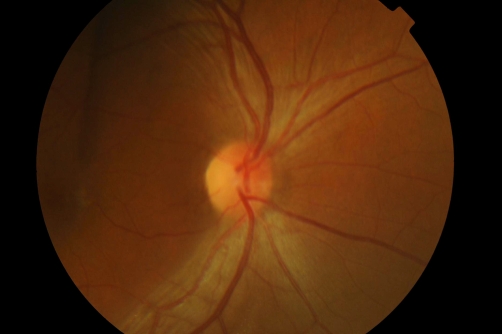
Slit lamp view at presentation.

**Figure 2.**
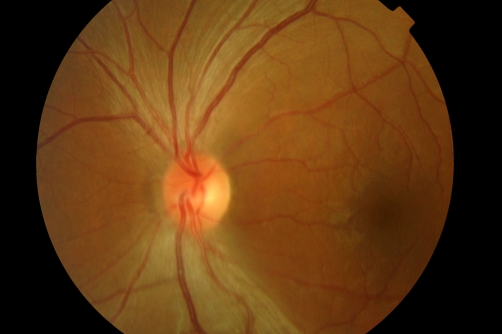
Bilateral optic disc pallor.

## Conclusion

Strachan's syndrome incorporates a spectrum of symptoms and signs and its aetiology remains a source of continuing debate. To our knowledge, this is the second report of such a syndrome occurring in the United Kingdom, in this case highlighting the association between optic and peripheral neuropathy, poor diet with associated vitamin deficiency, and previous residence in a tropical or sub-tropical climate. The extent to which these factors contribute to the disease is yet to be fully determined.

## List of abbreviations

ACE: Angiotensin-Converting Enzyme
APTT: activated partial thromboplastin time
BMI: Basal metabolic index
CRP: C-reactive protein
EMG: Electromyography
ENA: Extractable nuclear antigens
ESR: Erythrocyte sedimentation rate
LHON: Leber's hereditary optic neuropathy
